# Metastatic carcinoma of the cervix with unexpected ovarian involvement: A rare case report and review of the literature

**DOI:** 10.1016/j.ijscr.2025.111605

**Published:** 2025-07-03

**Authors:** Soheila Aminimoghaddam, Niloufar Sarchami, Marzieh Mohammadi

**Affiliations:** a Department of Obstetrics and Gynecology, School of Medicine, Iran University of Medical Sciences, Tehran, Iran; b Department of Obstetrics and Gynecology, School of Medicine, Iran University of Medical Sciences, Tehran, Iran; c Department of Obstetrics and Gynecology, Iran University of Medical Sciences, Tehran, Iran

**Keywords:** Cervical adenocarcinoma, Ovarian involvement, Positron emission tomography scan

## Abstract

**Introduction and importance:**

Ovarian metastasis from cervical carcinoma (CC) is rare. As adenocarcinomas tend to develop within the endocervix, they may be hidden in Pap tests and have a normal Colposcopic appearance. Only a limited number of Ovarian metastases from CC are reported in the literature, and various aspects of this involvement still need to be clarified.

**Presentation:**

In this study, we reported a case of unexpected ovarian metastatic cervical cancer in a 57-year-old female. The uniqueness of the present case was the large size of the metastatic ovarian mass, which led to ureter compression from the AC and the good response to chemoradiotherapy. Unlike the previously reported cases in which the ureter was obstructed at the ureter-bladder junction(trigone), in our case, ureter obstruction occurred at the level of the pelvic brim.

**Clinical discussion:**

Even though ovarian metastasis of cervical cancer is rare, chemoradiotherapy (CRT) manifests a good response with no remnant cervical mass observed in the surgery, which is compatible with the result of a normal Positron emission tomography scan (PET).

**Conclusion:**

Cervical adenocarcinoma can often be diagnosed at very late stages, presenting with large ovarian masses and symptoms of ureter compression. This highlights a lack of awareness regarding appropriate cervical cancer screening tests. Moreover, there is a need for appropriate following imaging methods to differentiate pelvic masses and lymph nodes.

## Highlights

• A metastatic carcinoma of the cervix was observed with unexpected ovarian involvement.
• Unlike the reported cases where the obstruction occurred in the trigone, the obstruction occurred in the ovary.
• The patient had a good response to chemotherapy (CRT).

## 1 Introduction

Cervical carcinoma ranks as the third most common gynecologic neoplasm worldwide. Although (cc) is preventable, in developing countries, it is still the second most frequent cause of cancer death [[1], [2], [3]]. The most common histologic type of cervical cancer is squamous, but the incidence of adenocarcinoma, with an endophytic growth pattern, is increasing. HPV is the causative agent in both squamous and adenocarcinoma of the cervix. In 2018, a classification system for endocervical adenocarcinomas was established by the International Endocervical Adenocarcinoma Criteria and Classification (IECC) [1]. This classification system distinguishes endocervical adenocarcinomas as either HPV-associated adenocarcinomas (HPVA) or non-HPV-associated adenocarcinomas (NHPVA) [2]. Patients with NHPVA display different clinical symptoms from those with cervical squamous cell carcinoma, and cervical cytological testing has a high false-negative rate, which might lead to misdiagnosis [3,4]. Previous investigations have indicated that NHPVA patients have larger tumor sizes and an unfavorable prognosis [5]. Although vaginal bleeding is the most common symptom in patients with cervical cancer, patients with advanced disease may present with obstructive uropathy.

Cervical cancers rarely metastasize to the ovaries. The proportion of cases presenting with ovarian metastases at the time of surgery ranges from 0.6 to 1.5 % [6,7]. Adenocarcinomas are more likely to metastasize to the ovaries than squamous cell carcinomas (SCCs). In different case series, 5–8 % of cervical adenocarcinomas *vs.* 0.4–1.3 % of SCCs metastasized to the ovary [8]. In this report, we present a patient with a massive rare ovarian involvement of cervical adenocarcinoma, which primarily presented with flank pain and hydronephrosis. The patient underwent chemoradiotherapy. In the 3-month follow-up imaging studies (PET-CT), complete cervical mass remission with no distant metastasis and a suspected solid pelvic mass or lymph node were reported. According to a radio-oncologist consult, the patient underwent surgery, which revealed an unexpected tumoral ovarian involvement.

## 2 Methods

The work has been reported in line with the SCARE criteria [9]. Written informed consent was obtained from the patient for the publication of this case report and the accompanying images. A copy of the written consent is available for review by the Editor-in-Chief of this journal on request.

## 3 Case report

**Fig. 1:**
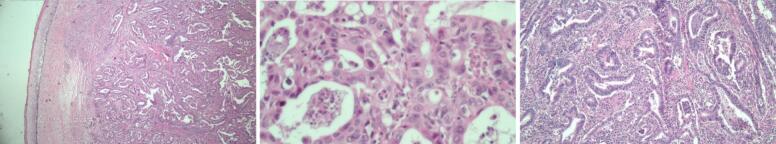
Pathological findings confirming HPV associated adenocarcinoma in the sample before surgery.

A 57-year-old menopaused G1P1L1 with no history of personal and familial cancer and no history of smoking and alcohol consumption primarily complained of right flank pain 24 months ago. After primary clinical evaluation, a vast 9 cm cervical mass and a 4 × 5.5 × 6.5 cm right pelvic mass at the level of pelvic brim, which had compressive pressure at the level of hydronephrosis, were reported by MRI and Positron emission tomography (PET). After double J insertion, a cervical mass biopsy was performed and sent for pathology. According to imaging and clinical studies, cervical cancer staging was IIIB. Pathology findings revealed HPV-associated adenocarcinoma (usual type) (Fig. 1).

**Fig. 2:**
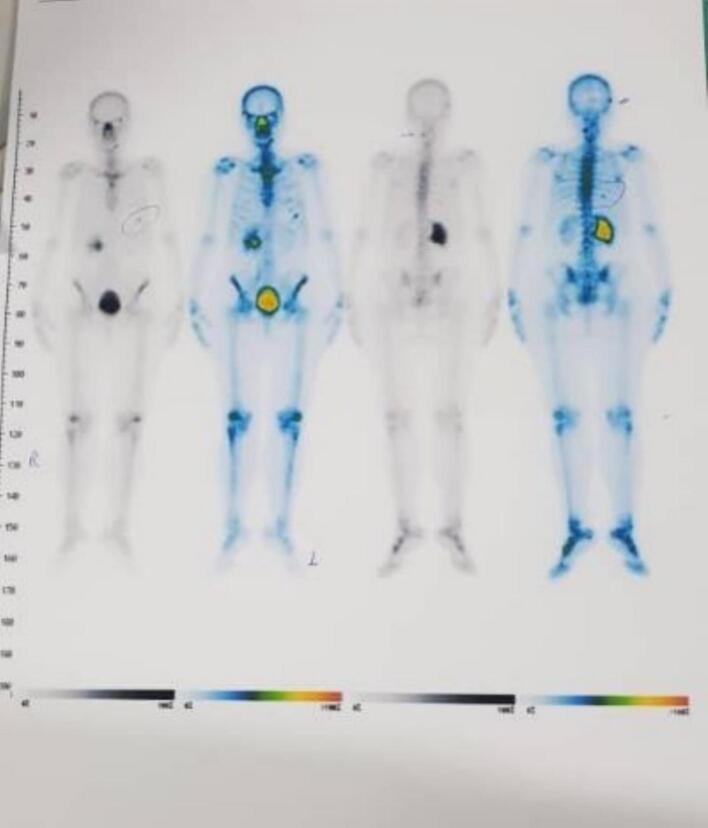
PET scan shows no other distant metastasis.

**Fig. 3:**
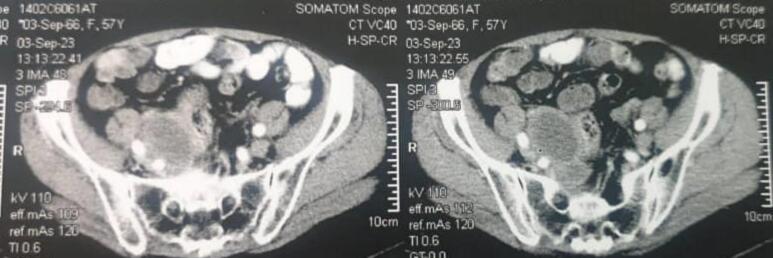
Preoperative radiographic findings.

According to tumoral clinical staging and tumoral pathology, the patient underwent Chemoradiotherapy (CRT) with cisplatin, which is superior to radiation alone for eight weeks (weekly). After rectum and bladder preparation, the patient underwent CT simulation. The patient also underwent pelvic MRI as fusion imaging of CT simulation. All simulation procedures were done in a single day. Simulation images were compared to diagnostic images of CT and PET scans. Arc therapy plans were independently constructed for Tomo therapy (Radixact9). The simultaneous integrated boost (SIB) plan was prescribed, 5040 cGy and 5600 cGy in 28 fractions. Clinical target volume (CTV) 5040 cGy included Common illiac LN, Internal illiac LN, Obturator LN, Endocervical cavity and 1/2–1/3 upper vaginal canal. CRV 5600 cGy Included cervical tumor with right parameter involvement with 0.5 cm margins. Planning target volume (PTV) was defined as 0.6 to 0.7 cm margin expansion from CTV. Daily cone beam computed tomography (CBCT) was used to accurate the patient's position before each day of treatment. After completion of external beam radiation therapy, pelvic MRI was done, and the patient underwent brachytherapy for 3 weekly fractions. For each fraction, the patient was sedated, a tandem was placed inside the cervix and uterus, and then needles (8 needles) were placed through the template into paracervical tissues and residue. After fixation of tandem and needles, CT simulation was done for the patient and was fused with post EBRT MRI for contouring. HRCTV (high risk CTV) was defined as the entire cervix, proximal of vagina, and any macroscopic extension or parametrial involvement at the time of brachytherapy. IRCTV (intermediate risk CTV) was defined as HRCTV plus a 1 cm margin. The bladder, rectum, and sigmoid were contoured as organs at risk. The dose prescription to HRCTV was 700 cGy each fraction. Who had received external beam radiation therapy for 28 fractions and Brachytherapy for three weekly fractions. The patient had also received concurrent chemotherapy with 40 mg/m2cisplatin. Patients who received radiotherapy should be monitored closely to assess treatment response. Cervical tumors may be expected to regress for up to 3 months after radiotherapy. Cervical or vaginal assessment should be performed every 3 to 6 months for 2 years [10]. Patients with isolated recurrences may benefit from surgical resection [10]. Although CT, PET, or IVP is not a part of routine post-radiotherapy surveillance, it should be performed if a pelvic mass is detected. This patient had no complaints after chemoradiation, but she was re-evaluated three months later. Although complete cervical tumor response and no other distant metastasis were seen on the PET scan (Fig. 2), due to an adnexal persistence mass in the CT scan findings (Fig. 3), the patient was referred for surgery by a radiation oncologist. Regarding final concurrent chemoradiotherapy, the response was categorized as partial response based on RECIST 1.1 [11].

An experienced gynecologic oncologist performed the surgery. The patient received enoxaparin before surgery and Keflin (2 g) immediately after anesthesia. Intermittent pneumatic compression (IPC) was also used for the patient during the surgery.

**Fig. 4:**
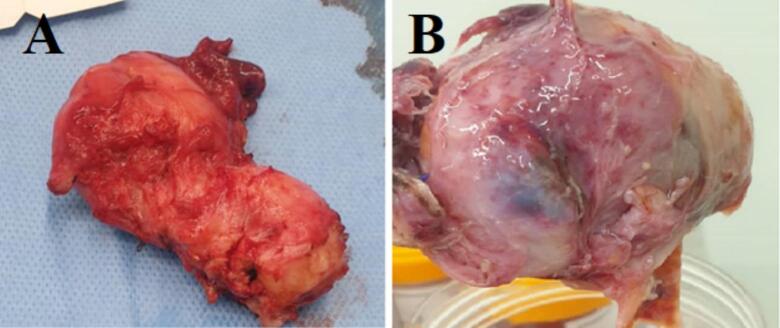
Photographs of the extracted mass. (A): cervix; (B): ovarian involvement.

**Fig. 5:**
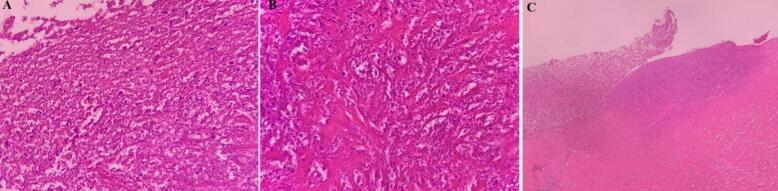
(a, b) Cervical pathology (×40); (b) in myxoid patterns, epithelioid tumor cells proliferated in loosely cohesive storiform arrangement (×10); (c) Necrotic ovarian wall (×40).

During surgery, a huge suspected tumoral right adnexal mass was seen, which may be metastatic from cervical cancer. The origin of the mass was the ovary, based on pursuing the Infundibulopelvic and utero-ovarian ligament. The mass was completely resected, and total hysterectomy with bilateral oophorectomy was performed cause the patient was in menopause, and we prefer not to reoperate the patient if the final pathology would be tumoral. Intraoperative, the extracted mass, which was cut by the surgeon, had no tumoral appearance (Fig. 3). This specimen was sent in the Formalin 10 % fluid for histologic and immunohistochemistry (IHC) evaluations (Fig. 4). It takes 10 min to perform fixation after specimen extraction, and the pathologist analyzes the specimen within 24 h. As the whole extracted mass was necrotic and no viable tissue was visible, IHC has not been performed by the pathologist. In IHC procedure, viable tissue is required to interact with the antibodies. Nonetheless, the shadow cells confirmed the tumor in the final pathology findings (Fig. 5), consistent with PET scan findings. As the final pathology revealed necrosis, there was no tumor antigen to perform IHC. Thus, IHC has not been performed.

After surgery, the patient received ceftriaxone (1 g/BD) and metronidazole (500 mg / BD) for 24 h. The patient also received enoxaparin (40 g/daily) for 3 days (until discharge). After discharge, the patient received metronidazole (500 mg /BD for 5 days) and apixaban (2.5 mg /BD) for 28 days. The patient was followed up with every three months for 24 months. The patient is still disease-free at 24 months after the surgery in the systemic examination. Also, no complication was recorded during the 24-month follow-up of the patients.

## 4 Discussion

Cervical adenocarcinoma accounts for 10–25 % of CC [12]. Both SCC and AC have unique features in their etiology, screening effectiveness, and prognosis. HPV infection is the leading cause of SCC and AC. A higher percentage of AC cases are the result of HPV 18 infections, especially among younger women [12]. Studies have shown that the incidence of Cervical adenocarcinoma, which arises from the cervical canal, is increasing worldwide [13]. As a result, almost 20 % of patients do not exhibit any symptoms during the early stages [14]. According to reports, the thin-prep cytologic test (TCT) has a 34.8 % rate of missed diagnosis in patients with cervical adenocarcinoma. In comparison, the high-risk HPV test has a negative rate of 17.7 %. This makes it challenging to identify the disease through screening promptly [15].

We reported a metastatic case of adenocarcinoma of the cervix with ovarian involvement, who primarily presented with flank pain and hydronephrosis. In advanced stages of cervical cancer, compressive hydronephrosis may be one of the notable late symptoms [16]. Especially in unscreened pap-tests women [17,18]. In the case reported in our study, the patient has not been referred for any screening tests for cervical cancer such as a pap test or HPV test. Since metastatic adenocarcinoma with ovarian involvement is rare, several aspects are still unconfirmed, and the number of available reports is very limited. Ngamcherttakul et al. reported two cases of ovarian metastasis from the cervix [19]. Both patients were over 60 years old and postmenopausal and had cervical cancer in the macroscopic stage IB1. In terms of histology, they were from SCC. The size of the cervical tumor was 2 cm in one patient and 3 cm in the other patient. The degree of ovarian involvement in both cases was much lower than in our case. Unlike these two cases, the tumor type in the study was adenocarcinoma, and the primary cervical tumor was much larger. In another study by HS Wu et al. [20], examining 1507 patients with CC, reported that ten patients had ovarian metastasis (6 patients were SCC, and four patients were AC, and the mean age of the patients was 45 years. In the most extensive available review, Landoni et al. [7] reported ovarian metastases from cervical cancer in 16 of 1695 patients, and the incidence rate was 0.9 %. They showed that older age of 45 years, higher FIGO stage or tumor size greater than 4 cm, histology SCC involved stromal, thickness of more than 3 mm, vascular, lymphatic space involvement, positive lymph node status [10], and lack of screening is the most important risk factors of ovarian metastasis. M Shimada et al., by examining 3471 patients with stage Ib to IIb cervical cancer who underwent radical hysterectomy, reported an ovarian metastasis rate of 1.5 % (52 patients). 6 people were in stage Ib1, 12 people were in stage Ib2, five people were in stage IIa, and 29 people were in stage IIb. The mean age was 49.9 years. The incidence of ovarian metastasis in patients with CC was 0.22 % for stage I, 0.75 % for stage IIa, 2.17 % for stage IIb with SCC, and 3.72 %, 5.26 %, and 9.85 % for adenocarcinoma, respectively. The survival rate was lower in the advanced stages, indicating the need for screening in women over 40. In most patients, the response rate to chemoradiotherapy was low [7,21]. Still, in our case, the response to treatment for the cervical tumor was very good, while the response rate to treatment for the pelvic mass was low. The difference between our case and the cases reported in the literature was the location of ureter obstruction; in previous cases, the junction of the ureter with the bladder (trigone) was blocked in cervical masses, while in our case, it was at the level of pelvic brim.

Another key finding of ours was the high accuracy of PET in diagnosing metastatic ovarian tumors from cervical cancer. In our case, the findings of PET were consistent with the final pathological findings in terms of the diagnosis of a completely necrotic tumor, which indicates the high accuracy of PET in the diagnosis of cervical tumors and metastatic tumors from the cervix. In 2018, the International Federation of Gynecology and Obstetrics (FIGO) staging system added imaging methods and pathological information to diagnose and determine the final stage of cervical cancer. They showed that the new staging has high accuracy for classifying cervical cancer [22].

The literature review shows that cervical cancers that metastasize to other organs, including the ovary, may be diagnosed very late in advanced stages. In addition, they are still menopausal women in their late middle age who are neglected for routine cervical cancer screening tests, which shows the vital need for regular screening of women for cervical cancer and routine gynecologic examination.

The uniqueness of the present case was the large size of the metastatic ovarian mass involvement of the cervical cancer. Also, in our case, unlike the reported cases where the obstruction occurs at the junction of the ureter and the bladder, the obstruction occurred at the pelvic brim beside the ovary. The PET findings were completely consistent with the final extracted adnexal mass pathology result which is tumor necrosis that shows chemoradiation response. Unlike previous reported cases, the tumor had a very good response to concurrent chemoradiotherapy, and no cervical cancer mass was observed during surgery. The large size of the primary cervical lesion can be attributed to a lack of screening [18]. In our study the primary cervical biopsy verified the adenocarcinoma and the ovarian mass shadow cell characterization revealed the tumoral involvement. Although IHC has not been performed due to tumor necrosis, if some viable tissue has been remained after chemoradiotherapy the IHC could be more advantageous.

## 5 Conclusion

Finally, the diagnosis of metastatic ovarian tumor from cervical adenocarcinoma by CT scans alone is not recommended. Moreover, PET scans based on a new classification [22] have high accuracy in follow-up programs of cervical cancer. Therefore, more awareness and the use of radiographic methods are needed to distinguish metastatic adnexal masses. Interestingly, standard concurrent chemoradiation in advanced stages of cervical adenocarcinoma may have good responses.

## CRediT authorship contribution statement

SA: Writing – original draft, Data curation. SA, MM, NS: Writing – original draftd: Data curation. SA, NS and MM: Conceptualization, Data curation, Supervision, Writing – review & editing MM: Conceptualization, Data curation, Supervision, Writing – review & editing.

## Consent

Written informed consent was obtained from the patient for publication of this case report and accompanying images. A copy of the written consent is available for review by the Editor-in-Chief of this journal on request.

## Ethical approval

This case report was approved by the Ethics Committee of our institute.

## Guarantor

Soheila Aminimoghaddam.

## Sources of funding

No funding was provided for the completion of this manuscript.

## Research registration

NA.

## Declaration of competing interest

There are no conflicts of interest to disclose.
